# Comparison of blended e-learning and face-to-face-only education for resuscitation training in German schools – A cluster randomized-controlled prospective study

**DOI:** 10.1016/j.resplu.2024.100767

**Published:** 2024-09-13

**Authors:** Wolfgang A. Wetsch, Nikolas Link, Niels Rahe-Meyer, Rico Dumcke, Jan M. Stock, Bernd W. Böttiger, Sabine Wingen

**Affiliations:** a University of Cologne, Faculty of Medicine, Albertus-Magnus-Platz 1, 50931 Cologne, Germany; b University Hospital of Cologne, Department of Anaesthesiology and Intensive Care Medicine, Kerpener Str. 62, 50937 Cologne, Germany; c Franziskus Hospital Bielefeld, Department of Anaesthesiology and Intensive Care Medicine, Kiskerstraße 26, 33615 Bielefeld, Germany; d Bielefeld University, Faculty of Biology, Universitätsstrasse 25, 33615 Bielefeld, Germany; e L2R GmbH, Cliev 4, 51515 Kuerten, Germany; f FOM University of Applied Sciences, Agrippinawerft 4, 50678 Cologne, Germany

**Keywords:** Schoolchildren, KIDS SAVE LIVES, Blended learning

## Abstract

**Background & Objectives:**

Cardiopulmonary resuscitation (CPR) is the key for surviving cardiac arrest. Recent recommendations propose that CPR can – and should –be taught to schoolchildren. This e-learning-based study analyzes whether face-to-face CPR training can be partly substituted with e-learning by measuring CPR knowledge and self-efficacy in trainees.

**Methods:**

In this cluster randomized-controlled prospective, students attending grades 5 to 7 of a German secondary school volunteered to participate and were randomly assigned to one of two groups with different methods for CPR training each: a traditional instructor-led group (control) where students received face-to-face teaching by a BLS instructor (45 min), and an e-learning group (intervention) where schoolchildren were able to accomplish their theoretical CPR training using an e-learning module (15 min). CPR knowledge and self-efficacy were measured and compared before (t0) and after (t1) the training using questionnaires. Face-to-face CPR training (45 min) on manikins proceeded in both groups hereafter. The formal hypothesis was that e-learning would result in better CPR knowledge.

**Results:**

Overall, 375 students participated; 33 of which had to be excluded. 342 participants were included in statistical analysis (instructor-led group *n* = 109; e-learning group *n* = 233). The study was terminated early due to the Covid19 pandemic, and did not reach the required number of participants. Lacking statistical power, an analysis of the existing datasets failed to show superiority of e-learning vs. conventional training for CPR knowledge (*p* = 0.306). Both groups improved CPR knowledge (*p* < 0.001) and self-efficacy (*p* < 0.001) after CPR training and showed an equal, high level of satisfaction with their perceived training method (face-to-face: 4.1[4.0–4.2] vs. e-learning: 4.0[3.9–4.1]; *p* = 0.153; maximum 5 points).

**Conclusions:**

This study failed to demonstrate superiority for e-learning but was terminated early and hence underpowered. Further research is necessary to prove the efficiency of e-learning tools for CPR.

## Background

Sudden cardiac arrest poses an immediate risk for patients’ lives, and it remains a leading cause of death in industrialized nations.^1^, ^2^ Despite resuscitation research and medical progress, mortality rates remain high throughout the world,^3^ in both physician- and non-physician-manned EMS systems.^4^ Meaningful efforts have been taken since today to improve the probability of survival, e.g. the regularly published scientific recommendations on cardiopulmonary resuscitation (CPR) from the international liaison committee on resuscitation (ILCOR),^5^, ^6^ which become translated into nationwide resuscitation guidelines.^7^, ^8^ During the last years, evidence has become clearer that early basic life support (BLS) is a major key for possible survival of cardiac arrest, which has also led to a modified “chain of survival” especially highlighting the early links.^9^ Despite nationwide deployment of professional EMS with highly trained personnel, patients suffering from out-of-hospital cardiac arrest (OHCA) have little odds of survival if bystanders do not initiate BLS and instead just wait for EMS arrival. EMS can take 8–10 min to arrive on scene even in highly-developed and densely-populated areas,^10^ and if there is no blood flow during this time, the brain sustains irreversible damage. Thus, every effort must be taken to motivate bystanders to initiate CPR.^11^

Currently, the lay resuscitation rate in Germany is 51.3%,^12^ while in Scandinavian countries it is as high as 70–80%.^10^ One intervention that has been recommended worldwide and is likely to improve survival of cardiac arrest is to teach and train schoolchildren in CPR.^13^, ^14^, ^15^, ^16^, ^17^ CPR-training in schoolchildren is also recommended by ILCOR^18^ and the World Health Organization.^14^ However, the implementation of teaching CPR to schoolchildren has several burdens that need to be overcome.^19^ One limiting factor is that there are not enough CPR trainers, so a common approach to solve this problem has been to train teachers in CPR, and – as multipliers – the teachers teach their pupils.^20^ CPR training courses are highly standardized worldwide, as they want to give state-of-the-art treatment for cardiac arrest.^21^

E-learning has become a regular part of our lives, and has successfully become another option for training in many areas.^22^, ^23^, ^24^ E-learning can offer relatively low-cost, highly standardized training concepts to a large number of individuals. With new and powerful e-learning technologies becoming available, it is important to evaluate these digital educational resources might also be a successful element for CPR training of schoolchildren. In our study, we thus aimed to analyze whether face-to-face training can be substituted with e-learning by evaluating CPR knowledge and self-efficacy in schoolchildren. We hypothesized that e-learning would lead to better CPR knowledge in schoolchildren compared to conventional face-to-face training.

## Material and methods

### Study registration

This prospective, cluster randomized, controlled trial was conducted in accordance with the Declaration of Helsinki between August 2019 and October 2020. It was approved by the Ethics Committee of the University of Cologne (Head: Prof. R. Voltz; Approval no. 19-1241), and registered at the German Register for Clinical Studies (ID: DRKS00017707).

### Sample size calculation

The required sample size was estimated by the Institute for Medical Statistics, Informatics, and Epidemiology of the University Hospital of Cologne using a univariate ANOVA that compares the change in self-confidence (post minus baseline) between groups with a two-sided significance level of 0.05 and a power of 0.9. We assume a standard deviation of 2.6 based on the results of preliminary studies,^25^ equal group sizes, and a relevant mean change in the score of 2 points in the intervention groups compared to the control group. The sample size calculations were conducted using G*Power. To account for the cluster structure of the target variable, we multiply the sample size by a design effect of 2.2 (25 children per class and an assumed intra-class correlation of 0.05). This results in a required sample size of 225 (net) per group. With an estimated dropout rate of 20% over three months, the gross sample size was determined to be 270 per group (approximately 12 classes).

### Study population

Schoolchildren attending grades 5 to 7 at a secondary school in a suburban-to-rural area in North Rhine-Westphalia, Germany, were eligible to participate in our study. From all participating schoolchildren, written informed consent had been obtained from their parents or legal guardians. Since grades 5 to 7 were asked to participate; participating students were hence aged between 10 and 15 years. Exclusion criteria were missing consent from partents or legal guardians respectively.

### Randomization

Class-wise cluster-randomization of grades 5 to 7 to intervention or control group was performed using a randomization strategy provided by the Institute for Medical Statistics, Informatics, and Epidemiology of the University Hospital of Cologne; the classes attended served as a matching variable.

### Study design

Theoretical knowledge about CPR and self-efficacy in CPR competences were assessed by means of questionnaires at two timepoints: (t0) at the day of training immediately before the beginning, and (t1) immediately after the training has been finished. The original study design included a follow-up (t2) three months after the training, but due to school shutdowns during the Covid-19 pandemic, a third assessment had to be canceled.

We surveyed schoolchildrens’ demographic data (age, sex, and prior first-aid course participation) at t0. CPR knowledge was assessed with 12 items and a sum score was calculated for overall knowledge (maximum 12 points). In accordance to the “Check-Call-Compress” algorithm, which is promoted for the education of medical laypersons,^26^ questions about (1) correct checking for breathing & consciousness, (2) the correct number for calling emergency medical services (1 1 2), and (3) correct pressure-point and frequency were defined as high importance questions with a separate sum score calculation for the time-points t0 and t1 (maximum 6 points). Self-efficacy was measured with four items using a five-point Likert scale, ranging from (1) not confident at all to (5) very confident. Means per participant were calculated for a self-efficacy score. Additionally, participants were asked to rate their own satisfaction with the perceived training method (five items) after training on a 5-point-likert-scale ranging from (1) does not apply at all to (5) applies very much.

### Training concepts

The intervention group received an innovative e-learning CPR training course (15 + 45 min = 60 min) including a simulated CPR training video scenario and a supplemental CPR gaming scenario with theoretical and practical CPR tasks in the first-person-view. The e-learning concept was designed and provided by a professional e-learning company (L2R GmbH, Kuerten, Germany) according to medical and scientific specifications from our study team (University Hospital of Cologne, University of Bielefeld). After this 15-minutes interactive self-education module, schoolchildren received practical hands-on training (45 min) by a pre-qualified team of trainers. Control group received analogous conventional face-to-face CPR training (45 + 45 min = 90 min) as recommended by the Standing Conference of Ministers of Education in Germany. Face-to-Face training combined theoretical CPR education (45 min) presenting slides and the same 45-minutes practical hands-on CPR training session with individual resuscitation manikins as the intervention group. Practical training was designed completely identical in both groups, theoretical content and instruction was compliant to European and German CPR education guidelines.^21^ The training content included (i) function of the cardiovascular system, (ii) medical background to and epidemiology of OHCA, (iii) information about bystander CPR rates, (iv) detection of an OHCA, (v) emergency call, and (vi) chest-compression (hands position, compression frequency, and depth).

### Statistical analysis

For statistical analysis IBM SPSS Statistic Version 29 (IBM Corp., Armonk, NY, USA) was used. Participants were excluded from statistical analysis if they or their parents did not provide written consent of participation. Further, participants were excluded if they did not attend at both survey time points (t0;t1). Ordinary data were analyzed using the Mann-Whitney-*U* test. Binary data were analyzed using the Χ^2^-test. Statistical analysis of training efficiency from t0 to t1 within the groups was performed with *t*-test for dependent samples. For comparison of CPR knowledge, high importance knowledge, and self-efficacy between the face-to-face and the e-learning group Χ^2^-test was used at the two time-points (t0;t1). Statistical significance was accepted as a P value 0.05 or less.

## Results

Overall, 375 students volunteered to participate. Since the study had to be terminated early due to the Covid19 pandemic (resulting in lockdowns and homeschooling), the required number of participants to reach sufficient statistical power could not be achieved. Thirty-three students were removed from statistical analysis due to missing data (failure to attend both survey time-points t0 and t1). Finally, 342 participants were included into statistical analysis (instructor-led group *n* = 109; e-learning group *n* = 233). Median age of participants was 12 (IQR: 11–13; min. 10 years/max. 15 years). Of the participants, 51.5% (*n* = 176) were female and 48.5% (*n* = 166) were male, none specified non-binary; 58.6% (*n* = 198) reported a prior participation in a first aid course. Demographic characteristics are shown in Table 1.

**Table 1:** Demographic characteristics of participants.

**Characteristics**	**Overall**	**Face-to-face**	**e-Learning**	***p-value***
**Group (N ;[%])**	342 (100.0)	109 (31.9)	233 (68.1)	
**Sex**	*n* (%)	*n* (%)	*n* (%)	0.833^1^
*Female*	176 (51.5)	57 (52.3)	119 (51.1)	
*Male*	166 (48.5)	52 (47.7)	114 (48.9)	
**Age (Median[IQR])**	12 (11–13)	12 (11–13)	12 (11–13)	**0.002**^2^
*(n;[%]) 10*		10 (9.2)	19 (8.2)	
*11*		19 (17.4)	76 (32.6)	
*12*		31 (28.4)	76 (32.6)	
*13*		41 (37.6)	55 (23.6)	
*14*		7 (6.4)	7 (3.0)	
*15*		1 (0.9)	./.	
**Previous first-aid course** **N = 338**				0.178^1^
*yes (n;[%])*	198 (58.6)	71 (65.7)	127 (55.2)	
*no (n;[%])*	140 (41.4)	37 (34.3)	103 (44.8)	

IQR = Interquartile range.

1 x^2^-test between face-to-face and e-learning group;

2 Mann-Whitney-*U* test between face-to-face and e-learning group.

### CPR knowledge

E-learning was not superior, as there was no significant difference between the two groups concerning knowledge about CPR both before (*p* = 0.094) and after training (*p* = 0.306). Additionally, knowledge score regarding high importance questions “Check-Call-Compress” failed to show superiority for e-learning (*p* = 0.906 before and *p* = 0.227 after training). Overall, each training had a significant effect in raising both overall and high-importance knowledge in all groups (*p* < 0.001 each) (Table 2).

**Table 2:** Group-comparison of CPR knowledge (min. 0 points, max. 12 points), CPR knowledge “high importance questions” (i.e., emergency phone number, check for breathing, correct compression point, correct compression frequency,; min. 0 points, max. 6 points) and self-efficacy before (t0) and after (t1) face-to-face or e-learning theoretical BLS training.

	**Overall** Mean [95 % CI]	**Face-to-face** Mean (95 % CI)	**E-Learning** Mean (95 % CI)	***p-value***^1^
**CPR knowledge at baseline (t0)**	6.9 [6.7–7.1]	7.1 [6.8–7.4]	6.8 [6.6–7.0]	*0.094*
**CPR knowledge after training (t1)**	8.1 [7.9–8.2]	8.1 [7.9–8.3]	8.1 [7.9–8.2]	*0.306*
***p-value***^2^	***<0.001***	***<0.001***	***<0.001***	
**CPR knowledge high importance questions at baseline (t0)**	3.7[3.6–3.8]	3.7[3.5–3.9]	3.7[3.5–3.8]	*0.906*
**CPR knowledge high importance questions after training (t1)**	4.8 [4.8–4.9]	4.8 [4.7–4.9]	4.9 [4.8–5.0]	*0.227*
***p-value***^2^	***<0.001***	***<0.001***	***<0.001***	
**CPR self-efficacy at baseline (t0)**	3.9 [3.8–3.9]	3.8 [3.7–3.9]	3.9 (3.8–4.0)	*0.237*
**CPR self-efficacy after training (t1)**	4.2 [4.1–4.3]	4.3 [4.2–4.4]	4.2 [4.1–4.3]	*0.099*
***p-value***^2^	***<0.001***	***<0.001***	***<0.001***	
**Satisfaction with CPR training method**	4.0 [4.0–4.1]	4.1 [4.0–4.2]	4.0 [3.9–4.1]	*0.153*

1 x^2^-*t*-test between intervention and control group.

2 t-test between t0 and t1.

### Self-efficacy in performing CPR

Self-efficacy about CPR showed no significant differences between the groups, and increased in each group after training (Table 2).

### Satisfaction with the perceived training method

In general, schoolchildren showed a high level of satisfaction with their individual perceived training method. No difference between the two training methods was detected (Table 2).

## Discussion

In this study comparing e-learning to conventional CPR training in schoolchildren aged 10–15 years, e-learning failed to prove superiority over conventional training. Unfortunately, the study was conducted at a time where the Covid19 pandemic started, resulting in halting the study without having included the required number of participants and hence with insufficient statistical power. As repetitive lockdowns, home-schooling, and restrictive federal regulations hindered recruiting the missing participants for a long time, we decided to analyze and publish our data despite being “incomplete”, since we deemed the results highly important and we did not want to include new participants with a break of more than two years.

This underlying study revealed several findings: First, e-learning was not superior concerning CPR knowledge; training results between face-to-face and e-learning education were comparable, although the e-learning training session was 30 min shorter than conventional instructor-led-CPR education. Second, e-learning based CPR training in 10–15 year-aged schoolchildren results in improved training outcomes regarding knowledge about resuscitation performance, knowledge about the most important steps “Check-Call-Compress”, and perceived self-efficacy in performing CPR. Third, schoolchildren showed a high and equal level of satisfaction with their perceived training method.

This is the first comparative e-learning study for CPR education in a German school setting. Our findings are in line with international published data in this field, which show comparable learning outcomes between traditional instructor-led CPR training and online-based (e-learning) training methods.^27^, ^28^ E-learning training sessions led to a standardization of training contents and support high quality education in this area.^28^ As shown by Teague et al., online-based only CPR education did not result in adequate practical skills and should be combined with practical hands-on training for best practical CPR skill performance.^29^ Since in Germany CPR education in schoolchildren have not been implemented nationwide although statutory recommendation for CPR training in schoolchildren exists since 2014,^17^ e-learning could foster schools engagement in this area. Educating schoolchildren in CPR has proven to be highly efficient, but is very demanding in terms of costs and personnel.^30^ Shown by our study, an abbreviated CPR training combining an interactive self-education tool and practical hands-on training could be a cost-and time-effective solution for CPR training in schools. Moreover, changes in resuscitation guidelines and standardized training content, addressing the helpers’ sex, for instance, can effectively be integrated into e-learning modules.^30^ Since a minority of interviewed teachers feel competent to serve as multipliers for CPR training in schools and to teach CPR,^31^, ^32^ e-learning could play a beneficial role as it offers prepared, standardizes, theoretical CPR training content. Subsequently, blended learning provides the opportunity to teachers, to rely on proven information and to reduce their own responsibility when teaching medical contents.^31^ With respect to the schools’ curricula and limited time resources for BLS teaching, another pedagogical advantage of e-learning is the flexibility of learning^33^:

• The learners are engaged to proceed in their own specific pace (e.g. pause or repeat specific parts)
• Asynchronous formats can be outsourced during non-teaching hours and offer an alignment of knowledge in advance (e.g before hands-on BLS activities)
• Since learning takes place individually, e-learning supports the learners’ self-regulation abilities in learning; however, if students are not motivated, e-modules could be left unfinished by certain learners.

A beneficial role of this approach has been reported even for low-resource countries.^34^ Unpaid online-courses educating CPR to schoolchildren and to other medical laypersons have the potential to reach those who have no way to attend BLS training in classrooms.^35^ However, as shown by Birkun et al., there is a need for a better quality of existing online BLS courses regarding international CPR guideline-conformity.^35^ Since schoolchildren are target group for online-learning, the integration of e-learning-elements in the “KIDS SAVE LIVES”-initiative^15^ is a promising approach to bring resuscitation knowledge into the German classrooms and beyond.

In the long term, e-learning with open educational resources (OER) also meets the demand for digitalization in the educational system and promotes students' digital skills as established by the UNESCO or the German Conference of the Ministers of Education (KMK), for instance.^36^, ^37^

## Limitations

Originally, the study plan includes more schoolchildren and a follow-up of CPR skills after three months, which could − due to COVID-19 pandemic − not be implemented. Since not all school classes could be enrolled as planned, the numerous and age differences between both conditions – described above – exist at the beginning. In our study, e-learning did not leave to superior but to comparable knowledge about CPR compared to conventional face-to-face education. These findings underline the efficiency of our developed innovative CPR training approach for schoolchildren with different prior levels of learning. Moreover, it has to be noticed that questionnaire-based learning outcomes like CPR knowledge have a limited statement regarding the schoolchildren’s behavior in a real-case emergency (“intention behavior-gap”).^38^

Further it has to be mentioned, that another barrier in implementing digital media and e-learning tools in schools is the cautious attitude and moderate application through teachers in their pedagogical planning^39^, ^40^ as well as the availability of OER.^41^ These circumstances were not addressed within this controlled trial and may need further investigation.

## Conclusions

In conclusion, our study failed to demonstrate superiority for e-learning in comparison to conventional face-to-face training for CPR. However, since both methods showed comparable results despite the e-learning was significantly shorter, further research with adequate power might detect a difference and is hence highly needed.

## CRediT authorship contribution statement

**Wolfgang A. Wetsch:** Writing – review & editing, Writing – original draft, Conceptualization. **Nikolas Link:** Writing – review & editing. **Niels Rahe-Meyer:** Writing – review & editing. **Rico Dumcke:** Writing – review & editing. **Jan M. Stock:** Writing – review & editing, Software, Methodology. **Bernd W. Böttiger:** Writing – review & editing, Supervision, Resources, Project administration, Funding acquisition, Conceptualization. **Sabine Wingen:** Writing – review & editing, Writing – original draft, Visualization, Supervision, Software, Resources, Project administration, Methodology, Investigation, Funding acquisition, Formal analysis, Data curation, Conceptualization.

## Declaration of competing interest

The authors declare the following financial interests/personal relationships which may be considered as potential competing interests: Wolfgang A. Wetsch, Nikolas Link, Niels Rahe-Meyer and Rico Dumcke have no conflicts of interest to declare regarding this manuscript.

Jan Stock is Head of the L2R GmbH and Projekt Manager at the Hans Peter Esser GmbH.

Bernd W. Böttiger is treasurer of the European Resuscitation Council (ERC), Founder of the ERC Research NET, Chairman of the German Resuscitation Council (GRC), Member of the „Advanced Life Support (ALS) Task Force of the International Liaison Committee on Resuscitation (ILCOR), Member of the Executive Committee of the German Interdisciplinary Association for Intensive Care and Emergency Medicine (DIVI), Founder of the “Deutsche Stiftung Wiederbelebung”, Federal Medical Advisor of the German Red Cross (DRK), Member of the Advisory Board of the “Deutsche Herzstiftung”, Co-Editor of “Resuscitation”, Editor of the Journal “Notfall + Rettungsmedizin”, Co-Editor of the Brazilian Journal of Anesthesiology. He received fees for lectures from the following companies: Forum für medizinische Fortbildung (FomF), Baxalta Deutschland GmbH, ZOLL Medical Deutschland GmbH, C.R. Bard GmbH, GS Elektromedizinische Geräte G. Stemple GmbH, Novartis Pharma GmbH, Philips GmbH Market DACH, Bioscience Valuation BSV GmbH, Becton Dickinson GmbH, Fundacja Polski Instytut Evidence Based Medicine.

Sabine Wingen is executive personal assistant of the executive board of the German Resuscitation Council (GRC).
